# *Notes from the Field:* Identification of a *Triatoma sanguisuga* “Kissing Bug” *—* Delaware, 2018

**DOI:** 10.15585/mmwr.mm6815a5

**Published:** 2019-04-19

**Authors:** Paula Eggers, Tabatha N. Offutt-Powell, Karen Lopez, Susan P. Montgomery, Gena G. Lawrence

**Affiliations:** ^1^Division of Public Health, Delaware Health and Social Services; ^2^Delaware Department of Agriculture, Dover, Delaware; ^3^Division of Parasitic Diseases and Malaria, Center for Global Health, CDC.

In July 2018, a family from Kent County, Delaware contacted the Delaware Division of Public Health (DPH) and the Delaware Department of Agriculture (DDA) to request assistance identifying an insect that had bitten their child’s face while she was watching television in her bedroom during the late evening hours. The parents were concerned about possible disease transmission from the insect. Upon investigation, DPH learned that the family resided in an older single-family home near a heavily wooded area. A window air conditioning unit was located in the bedroom where the bite occurred. The family reported no recent travel outside the local area.

The insect was preliminarily identified as *Triatoma sanguisuga* (a “kissing bug”) by staff members from DDA. Triatomines are blood-sucking insects that feed on animals and humans, and they have a predilection for biting the faces of humans (*1*). DPH and DDA jointly contacted Texas A&M University’s Kissing Bug Citizen Science Program, a multidisciplinary research program aimed at documenting and collecting kissing bugs from across the United States.* The insect was identified based on a photograph as *Triatoma sanguisuga,* a vector that can transmit the protozoan parasite *Trypanosoma cruzi* which causes Chagas disease (*1*,*2*). Subsequently, the insect was sent to CDC, where species-level identification was morphologically confirmed. Conventional polymerase chain reaction testing of the triatomine hindgut was negative for *T. cruzi*. Bloodmeal analysis detected a human bloodmeal; the girl who was bitten had no ill effects.

This finding represents the first confirmed identification of *T. sanguisuga* in Delaware. Texas A&M had received a previous report of a suspected kissing bug in July 2017 from Kent County, Delaware. That insect was found dead with no reported human exposure. Photographic identification by Texas A&M indicated *T. sanguisuga;* however, physical inspection by a local institution in Delaware had initially identified it as a milkweed bug and destroyed it before the client contacted Texas A&M, precluding definitive identification.

Chagas disease can cause serious cardiac and gastrointestinal complications. CDC estimates that approximately 300,000 persons with Chagas disease live in the United States, and most were infected with *T. cruzi* in the parts of Latin America where Chagas disease is found. Triatomine bugs also are found in the United States, but only a few cases of Chagas disease from contact with the bugs have been documented in this country (*2*). Although presence of the vector has been confirmed in Delaware, there is no current evidence of *T. cruzi* in the state (*2*). *T. cruzi* is a zoonotic parasite that infects many mammal species and is found throughout the southern half of the United States (*2*). Even where *T. cruzi* is circulating, not all triatomine bugs are infected with the parasite. The likelihood of human *T. cruzi* infection from contact with a triatomine bug in the United States is low, even when the bug is infected (*2*).

Precautions to prevent house triatomine bug infestation include locating outdoor lights away from dwellings such as homes, dog kennels, and chicken coops and turning off lights that are not in use. Home owners should also remove trash, wood, and rock piles from around the home and clear out any bird and animal nests from around the home. Cracks and gaps around windows, air conditioners, walls, roofs, doors, and crawl spaces into the house should be inspected and sealed. Chimney flues should be tightly closed when not in use and screens should be used on all doors and windows. Ideally, pets should sleep indoors, especially at night, and outdoor pet resting areas kept clean. Finally, homeowners might consider using a licensed pest control professional for insect control (*1*,*2*).

## Footnotes

* https://kissingbug.tamu.edu/.
